# How do primary care consultation dynamics affect the timeliness of cancer diagnosis in people with one or more long-term conditions? A qualitative study

**DOI:** 10.1136/bmjopen-2025-103288

**Published:** 2025-09-28

**Authors:** Maria Valasaki, Mary Carter, Rachel Winder, Elizabeth Shephard, Jose M Valderas, Samuel William David Merriel, Leon Farmer, Beccy Summers, Sarah Gerard Dean, Sarah Morgan-Trimmer

**Affiliations:** 1 Public Health and Primary Care, University of Cambridge, Cambridge, UK; 2 Health & Community Sciences, University of Exeter, Exeter, UK; 3 University of Exeter Medical School, Exeter, UK; 4 National University of Singapore, Singapore; 5 Centre for Primary Care & Health Services Research, The University of Manchester, Manchester, UK; 6 PPIE, University of Exeter Medical School, Exeter, UK; 7 PenCLAHRC University of Exeter Medical School, Exeter, UK; 8 School of Primary Care, University of Southampton Faculty of Medicine Health and Life Sciences, Southampton, UK

**Keywords:** Primary Care, Chronic Disease, QUALITATIVE RESEARCH, Decision Making, Early Detection of Cancer

## Abstract

**Abstract:**

**Objectives:**

To explore how pre-existing conditions affect the diagnostic process for potential cancer in primary care patients.

**Design:**

Qualitative interview study using thematic analysis underpinned by a critical realist approach.

**Setting:**

Primary care practices recruited through four Clinical Research Networks and UK health charities across England.

**Participants:**

Interviews were conducted with 75 patients with one or more pre-existing conditions (anxiety/depression, diabetes, obesity, chronic obstructive pulmonary disease, Parkinson’s disease or multiple long-term conditions (four or more)) and 28 primary care professionals (general practitioners and nurses).

**Results:**

The study identified legitimacy as a central theme influencing patient trajectories in the health system while trying to receive a diagnosis for symptoms with which they presented to primary care. Patients engaged in self-triage to determine whether symptoms were ‘legitimate’ enough to seek care. Subsequent triaging steps (by receptionists, nurses and online systems) acted as gatekeepers, with decisions influenced by effectiveness of describing the symptom and subjective impressions. During consultations, clinicians relied on a mix of symptom narrative clarity, medical history and objective ‘metrics’ (eg, blood results, family history) to determine legitimacy for further investigations. Pre-existing conditions could either lower the threshold for referrals or obscure potential cancer symptoms. The stigma associated with mental health diagnoses often undermined perceived legitimacy and contributed to delays.

**Conclusions:**

Legitimacy is continuously negotiated throughout the diagnostic pathway. It is shaped by social, moral and biomedical judgements. To promote early cancer diagnosis for patients with pre-existing conditions, clinicians must make legitimacy assessments explicit, reduce stigma especially around mental health and standardise triage processes.

**STRENGTHS AND LIMITATIONS OF THIS STUDY**

- Interviews included a diverse sample of 75 patients and 28 clinicians across England.
- Analysis incorporated perspectives from patients with a range of pre-existing conditions and included strong patient and public involvement and engagement.
- Did not include demographic factors (eg, age, gender, deprivation) in the analysis.
- No comparison group of patients without pre-existing conditions was included.
- Often, the outcome of the consultation is not reported in the text.

## Introduction

 Cancer is one of the main causes of death worldwide. In the UK, while a slight decline in the mortality rate is projected by 2035, overall cancer cases and deaths are expected to increase. This is mainly due to increases in size and age of the population.^1^ Although timely diagnosis is crucial,^2^ complexities in primary care consultations,^3 4^ systemic, social and personal factors contribute to diagnostic delays of cancer.^5^ Several studies highlight that being from an ethnic minority,^6^ being old and frail,^7^ being a female,^8^ living in deprivation and/or having specific cancers,^9^ or having comorbidity^5^ are factors that are associated with delayed cancer diagnosis. Specifically, pre-existing long-term conditions appear to delay timely cancer diagnosis in primary care.^10^,^12^

Social and systemic factors contribute to diagnostic delays not only by impacting the consultation process but also by creating barriers to accessing care. The candidacy framework^13^ offers an in-depth explanation of accessing healthcare by referring to the dynamic process by which individuals are identified or self-identify as eligible for healthcare services. It involves both individual actions (seeking care, describing symptoms) and institutional responses (healthcare provider decisions, system-level policies). Research highlights how systemic factors such as limited appointments and time restrictions affect patients’ ability to express symptoms and to feel entitled to care, with doctors’ judgements influencing whether symptoms are taken seriously, especially when vague.^12^ Similarly, there are reports about patients being ‘unsuitable’ candidates for treatment and allocation of resources, raising concerns about the predominant biomedical paradigm.^14^ The concepts of candidacy and eligibility provide a framework to explain general practitioner (GP)–patient interactions and the processes patients go through when determining whether their symptoms are legitimate enough to justify a GP visit, often to avoid wasting GP’s time.^15^

In the healthcare context, candidacy is used to negotiate access, emphasising the roles of perception, interaction and systemic barriers. Legitimacy, as we shall explore in this article, aligns with the concept of candidacy, but also highlights the moral and social acceptance of a claim or status. The concept of legitimacy has been discussed in several studies, and in healthcare refers to the perceived validity of a patient’s condition or need for care, as judged by the patient themselves, healthcare providers and broader societal norms^16^,^18^; it is a socially constructed concept, grounded in processes of justification, recognition and categorisation and is deeply influenced by cultural, institutional and systemic factors.

In this article, we explore the process of deciding to visit the GP, the consultation itself, and the pathway to referral for further investigations in patients with pre-existing conditions who present with symptoms potentially indicative of cancer. The concept of legitimacy inductively emerged and, in this article, is used as an analytical lens to understand how patients’ concerns are framed, interpreted and acted on within primary care. Unlike much of the existing literature, which focuses on legitimacy in acute or emergency settings or in relation to medically unexplained symptoms, this article extends the concept to the diagnostic journey in primary care for patients with chronic conditions—highlighting how legitimacy is negotiated over time, shaped by medical histories and influenced by both patient self-perception and clinical judgement. Moreover, we explore legitimacy in relation to candidacy, concepts that our data reveal to be interconnected.

## Methods

As part of a larger research programme, the aim of which was to identify how to spot cancer early for people with comorbidities in primary care (SPOtting Cancer among Comorbidities), we conducted qualitative interviews with patients, GPs and primary care nurses across England to investigate their perceptions and experiences on how specific pre-existing conditions might affect cancer diagnosis in primary care. The pre-existing conditions or health status included patients with anxiety and/or depression or both, chronic obstructive pulmonary disease (COPD), obesity (body mass index ≥30 kg/m^2^), diabetes, multiple long-term conditions (four or more conditions) and Parkinson’s disease. These conditions were chosen as those that are potentially associated with higher risk of late cancer diagnosis, based on current literature.

### Recruitment

We used two recruitment routes. First, general practices were recruited via four Clinical Research Networks (CRNs). Participating practices ran one or two database searches to identify patients who had visited their GP in the past 12 months with a symptom potentially indicative of cancer, based on a symptom list developed by the team using SNOMED codes. Hand-searching was used to exclude patients (eg, severely unwell). Inclusion criteria were: age over 18, presence of a relevant pre-existing condition and a GP visit in the past year with a listed symptom. Exclusion criteria included: no recall of a recent GP visit, being identified by practice staff as unsuitable (eg, acutely unwell, communication difficulties, welfare concerns, lack of capacity or ‘decline contact’ flag). Eligible patients were sent an invitation letter, information sheet and consent form and asked to contact the research team if interested. GPs and nurses were recruited through CRNs contacting practices in their region. Inclusion criteria included at least 1-year experience in general practice and experience of consulting (face-to-face, online or by phone) with patients with relevant pre-existing conditions.

The second recruitment route—suggested by patient and public involvement and engagement (PPIE) members—was through UK charities, to boost participation due to lower-than-expected recruitment via practices. We contacted 23 charities; 5 advertised the study on their websites and social media. Interested participants completed eligibility questions and were directed to the research team. All participants received gift vouchers, and practices were reimbursed for service support and research costs.

### Interviews

Two interview guides were developed for the two groups, patients and clinicians (online supplemental appendix 1 and online supplemental file 2)) in collaboration with PPIE members and GPs and nurses from the Department of Health and Community Sciences at the University of Exeter. Two experienced non-clinical researchers in qualitative research conducted the interviews. Interviews with patients were conducted in person, over the phone or online by using Zoom; all interviews with GPs and nurses were conducted online or over the phone. All interviews were one to one and were recorded after receiving written consent from participants. For participants who could not provide written consent due to technological difficulties, oral consent was taken and recorded. Interviews were transcribed verbatim by external transcribers and uploaded into NVivo for analysis. All interview data and information about participants were stored on the University of Exeter secure servers to which only the immediate study team had access.

### Analysis

Analysis began after the initial interviews and recruitment for all groups stopped once sufficient information power had been reached.^19^ Continued recruitment was only pursued for patients with anxiety and/or depression, as emerging findings in the wider study warranted further exploration, resulting in a larger sample. Having completed the interviews, the research team first analysed interviews with clinicians and patients, grouping patient interviews according to their pre-existing conditions. The team then compared these condition-based groupings to identify similarities and differences in patients’ perceptions, views and behaviours. While each group exhibited some distinct characteristics, common themes emerged across all groups.

The analysis began at the start of data collection and was refined after joint analysis of several interviews. To enhance the trustworthiness of our study, coding was conducted, and a codebook was developed by three members of the research team to ensure dependability and reduce individual bias. In addition, the codebook was discussed and refined at multiple stages in collaboration with PPIE members, supporting the credibility and confirmability of the analysis through reflexive dialogue. For example, PPIE input helped clarify codes such as ‘decision-making for visiting the GP,’ which, alongside others, contributed to the development of the theme of legitimacy.

A combination of deductive and inductive approaches was employed. Deductive analysis was used to identify pre-existing mechanisms described in the literature^12^; researchers systematically coded data for these mechanisms (eg, the surveillance effect, perceived prior odds etc). In contrast, inductive analysis allowed for the emergence of new themes—such as legitimacy—without relying on predefined theories. We applied segment-by-segment ‘open codes’ without forcing them into pre-existing categories, capturing anything we found meaningful in the text. The team applied thematic analysis^20 21^ as the primary analytical method, adapting it as needed for the project. The analysis was underpinned by a critical realist perspective,^22^ enabling exploration of underlying social and psychological processes through the combined perspectives of clinicians and patients. This approach aligned well with the researchers’ social science backgrounds.

To ensure patients’ anonymity, we used numbers and the initials of their index condition, ANX: anxiety, A/D: anxiety and depression, DIA (diabetes), OBE (obesity), PD (Parkinson’s disease), COPD, MLCs (multiple long-term conditions), for example, ANX1, DIA3, COPD4, etc. For GPs, we used numbers after the initials that indicate that these participants are GPs, for example, GP1, GP2, GP3. For nurses, we did the same, for example, NS1, NS2 etc. Interview numbers are random and do not indicate the order in which the interviews were conducted.

### PPIE involvement

PPIE members were first involved in the research during the early stages of study development, contributing valuable insights into the research question. Their priorities, experiences and preferences significantly shaped the formulation of the research question, ensuring that it addressed real-world concerns relevant to the target population. In terms of study design and conduct, patients and public representatives played an active role by providing feedback on the topic guide. PPIE involvement extended to the recruitment process, where their perspectives were integral in shaping recruitment strategies that were accessible and appealing to potential participants. PPIE was also involved in the analysis by providing feedback on the development of codes and themes. Looking forward, PPIE will continue to be engaged in selecting appropriate methods for disseminating the study results. They will also play a key role in agreeing on plans to share findings with participants and broader communities, ensuring that the research is communicated effectively and meaningfully to those it aims to serve.

## Results

In total, we collaborated with 23 practices from four CRN areas, and each practice invited approximately 30–60 patients. Similarly, we collaborated with five charities. Table 1 provides details of the collaborating organisations and total numbers of participants.

**Table 1:** Sample frame results

	Patients						GPs	Nurses
**Area**	**Diabetes**	**Anxiety/depression**	**Obesity**	**Chronic obstructive pulmonary disease**	**Parkinson’s disease**	**Multiple long-term conditions (four or more**)	**x**	**x**
South West of England	7	18	10	8		3	12	9
North West London				1			1	1
West of England						6		
West Midlands	2						3	2
Charities	2	3	0	1	12			
Total	11	21	10	10	12	11	16	12
	75						28	

GP, general practitioner.

### Constructing legitimacy

In this article, we discuss results related to the theme of legitimacy, which inductively emerged as the strongest and most prevalent finding from all participants and groups. Additional findings will be reported elsewhere. We analysed how patients articulated a symptom and timeline of any help-seeking behaviour, alongside their descriptions of pre-existing conditions. We also analysed how GPs/nurses talked about patients and their symptoms by evaluating multiple factors (e.g., blood results, family history, behavioural patterns). We then critically interpreted this information in order to understand how patients identify themselves as legitimate and how GPs and nurses make decisions about patients’ legitimacy for investigating further for cancer. Table 2 presents the three subthemes of the main theme of legitimacy, accompanied by illustrative quotes from the interviews.

**Table 2:** Subthemes with illustrative quotes

Legitimacy	
**Subthemes**	**Quotes**
Pre-existing conditions	“‘Cos I think, you know, all this stuff about multimorbidity really does muddy the waters, unfortunately.”, GP7 “I think if somebody comes to me with a flare-up of their COPD that isn't settling, I think the decision of when to re-X-ray is really hard because they've got a known COPD, they've had an X-ray last year that was normal, they have a flare that isn't settling they've got no other symptoms other than a cough which is chronic for them, I think those are difficult decisions”, GP4 “Does the comorbidity affect your decision to investigate further?… I think so, yes. I think it gives me a lower threshold. If there’s something I’m a little “mm, not quite happy with this presentation”, then if there are extra complicated or complications to their health that would give me a lower threshold to refer into a system to get them looked at promptly, urgently, yeah”, NS1 “He can't refer you. You know, I've been asking for a few months now to be referred to the urology department of the hospital, because I'm getting bladder problems. And he just… you know, oh, and then changes the subject. I don't know what I've got to do to make him refer me to the hospital. I can't self-refer.”, OBE9 “Yes, I mean, I would say the hardest group are probably the ones that you would call the hypochondriacs who are down the surgery all the time with symptoms which are vague and that those are the hardest ones to try and sift the wood for the trees”, GP8
The steps of triaging	“I would say, probably, it(patient refers to Parkinson’s)stops me more than it helps me. Because I mean, certainly thinking about going to the GP, because I think myself that maybe I’m making a bit of a fuss about something and maybe it’s only my Parkinson’s.”, PD2 “For me as a clinician, I think the key is to listen. It’s really, really important to listen and pick up. Because we do a lot of telephone triage, we don't… and we don't see everybody. We lose out on those facial clues and the… the body language cues. So it… so it’s up to me to sort of try and ascertain what they're trying to say to me.”, NS3 “At my doctor’s it’s fine, they’ve got a new system where you go onto, I think it’s x [specific software used in general practices], and you put in lots of answers to questions, it’s just an AI thing, and eventually it comes up saying yeah you’ve got an appointment at 11 o’clock this morning, can you make that appointment yes or no, depending on the answers to your questions.”, A/D5 “I think that across the board there’s a huge variety of how patients communicate their symptoms. And I think within probably all medicine, but certainly within primary care, you’ve got your brilliant historians, who come in with bullet points of their symptoms, and a well-defined chronology, and you’re like “Oh brilliant, now I know exactly what I’m going to do with you”. And then you get your patient who sits down and says “I just don’t feel quite right”. GP2 “Those are the ones(patients with A/D)where it may not immediately come to our mind as well. So, it’s that kind of—and, you know, because of the two week wait system, very much fine-tunes our thinking. If the patient doesn’t mention that red flag symptom or doesn’t say it in a way that makes it sound like it could be a red flag symptom, you know, there are different ways you can say something. So, if it doesn’t sort of trigger that little thing in our heads to go “Oh, hang on, maybe that’s something I need to look into”, GP1
The ‘metrics’	“No, I mean, they’re [fatigue] such a vague symptoms, aren't they? That’s the thing. I guess it’s in combination with bloods, history. I don't do the two week wait criteria, but I'm obviously aware of it, so I would signpost him to the GP, and I have signposted a couple of people with weight loss and change in bowel, and they've been referred on a two week wait, and I see they've had a bowel cancer diagnosis”, NS2 “If it’s something new, so something they've never had before; something they’re clearly concerned about. They might be telling me they have a very strong family history of cancer; they might be telling me they've already had cancer five years ago, this could be a recurrence. So, any of those scenarios is going to make me think it needs more investigating,” NS4 “So, the things I always ask everybody is are they eating and drinking, what’s their weight doing, if they’re a smoker, alcohol, change in bowel habits and waterworks. I mean, I kind of go through those symptoms”, GP8 “(…)to cancer on the side of my mum mainly. But my dad, also once he had cancer(…)and in the(…)the most part of the family die of cancer but only one survived after a long time I thought he survive also but he died… (…)he saw me at the clinic two weeks ago, they do a mammogram, they do a(…)but they are going… they want to refer me to(…)because… to a breast family history clinic because the history of my auntie and my cousin… (…)Because I was very concerned, I have this lump, it’s(…). She said, “Yes, we decided to refer you, because we(…)or not.” It was very good, the nurse practitioner who examined me. Whenever I spoke to her, I think, with the family history and all that and things like that. She examined me a lot, [indeed] the breast.” A/D4

### Pre-existing conditions

One of the main questions that clinicians were asked was how they think pre-existing conditions might affect cancer diagnosis in primary care. Apart from drawing on the underpinning medical science that might indicate cancer, GPs talked about dealing with more than one pre-existing condition and the challenges they bring to decision making for further investigations. The phrase ‘muddy the waters’ was used by a GP to describe the difficulty multiple conditions bring when clinicians diagnose symptoms that could also be cancer. Decisions GPs need to make seem complex. To decide whether to investigate further or not, GPs rely on specific factors that indicate or not the likelihood of a patient to have cancer. Symptoms of COPD, for example, and specifically a cough, seem to mask symptoms of specific types of cancer, making the decision to investigate further, by using that is, X-ray, challenging. However, along with the symptoms comes time which is constantly under evaluation and seems to play a crucial role in the construction of a judgement.

Not only GPs, but also nurses are part of the decision-making, who suggest that having one or more pre-existing conditions lowers the threshold for further investigations; the existence of comorbidities makes them more cautious and proactive in seeking further assessment. From patients’ perspectives, being further investigated is a matter of GPs’ judgement towards their symptoms. Specific symptoms or conditions, as participants have suggested, are linked or not linked with specific sets of cancer. If a pre-existing condition does not match the specific sets of cancers, then further investigations will probably not take place. One of these conditions is anxiety/depression. Some clinicians call patients with anxiety/depression ‘hypochondriacs’. In these cases, sometimes GPs attribute symptoms to their anxiety. Although it is already reported that patients with mental illness are diagnosed with cancer later than patients without mental illness,^23^ it is also observed from these excerpts that clinicians find it hard to trust them and their symptoms. Unlike patients with other conditions, those with anxiety and/or depression are perceived to visit the GP more frequently, reinforcing the notion of them as ‘hypochondriacs’ and potentially reducing the level of clinical attention their concerns receive.

Overall, while some clinicians adopt a cautious approach, lowering the threshold for further investigations, others may attribute symptoms to pre-existing conditions, thereby delaying referrals. Patients with mental health conditions, particularly anxiety and depression, often encounter additional barriers. Clinicians may sometimes perceive them as overly concerned with their health, leading to scepticism from GPs and potential under-investigation. Similarly, patients with conditions perceived as unrelated to cancer may struggle to be referred for further assessments. The social and moral assessments of pre-existing conditions, mediate the decision-making process, setting legitimacy as an inviolable factor for being further investigated. It appears that pre-existing mental health conditions could jeopardise patients’ legitimacy towards further investigations.

### The steps of triaging

Triaging here, as emerged from patients’ and clinicians’ interviews, has two aspects (a) the way patients evaluate their symptoms and decide to visit the GP and (b) how they construct and communicate their story and symptoms to clinicians before and during the consultation. Patients, regardless of their pre-existing conditions, talked about visiting or not visiting the GP for symptoms that worried them. To make the appointment, they would first go through an internal process, a kind of self-evaluation, that would allow them to decide if the symptom was sufficiently valid or necessary to bother the GP. Regardless of the pre-existing condition they have, they first need to establish their symptom(s) as legitimate grounds for seeing a GP. By evaluating the symptom, they legitimise themselves and only then will they make an appointment.

Having legitimised themselves, patients aim at getting an appointment with the GP. To do this, they often may first have to be triaged by the nurse or the reception staff, and for passing triage, they have to describe their symptom(s) either over the phone or by completing an online form. Triage is part of the process which involves describing the symptom and listening to what the patient says. But important aspects of the communicational and triaging procedure can be left out, and these are the non-verbal clues. At the same time, completing the online form before they get an appointment is equally important. In both cases, triaging means the legitimisation of the patient and their access to healthcare.

Having made the first step, patients often have to explain themselves and their symptoms once more, now during the consultation. GPs state how different this can be between patients and clearly explain the importance of effectively describing a symptom and how this is linked to their decision-making for further investigations. Patients need to narrate their stories effectively; effective description of a symptom is about being precise about its characteristics and the chronology; when a symptom persists, it becomes more legitimate for further investigations. Being able to describe a symptom in a way that will trigger clinicians to think of red flag symptoms will probably ensure further investigations. But the same does not apply to anxiety/depression, as previously explained.

### The ‘metrics’

Before they make any decisions for further investigations, GPs take into consideration multiple factors such as those we have described above; symptoms and their association with any pre-existing condition, association of the pre-existing condition with sets of cancer, effectiveness of describing the symptom and duration of the symptom. However, there are also ‘metrics’ that clinicians take into consideration before deciding to investigate further. These metrics (or objective measures), as GPs themselves call them, are often biologically oriented and can provide useful information for a patient’s health status. ‘Metrics’ is used here to refer to factors such as blood test results, family history, medication history and weight. Blood results or counting other biological indicators allow GPs, in accordance with the other factors, to decide when a symptom, that is not clearly red flag but could potentially be cancer, should be further investigated.

Along with bloods, family history is an indicator that will allow a clinician to make the decision to refer for further investigations. Although in this context family history matters for cancer risk, it raises concerns around knowledge of facts of cancer in the family (type of cancer, age of death etc), and relations between members of the family. Apart from family history and blood results, there are other biological factors that clinicians take into consideration which might indicate cancer risk. Patients’ age, sex, drinking or smoking status and weight affect how clinicians assess the risk of cancer and determine if a patient is legitimate for cancer investigations.

At the same time, patients narrate their stories through which clinicians’ decisions are explained. When clinicians observe objective factors such as family history of cancer, and when patients narrate their stories and describe their symptoms clearly and precisely, the latter become legitimate and further investigations take place. Such metrics provide a clinical foundation to legitimise both patients and clinicians; metrics certify patients’ legitimacy and clinicians’ choice to act further.

## Discussion

In this study, the concepts of legitimacy and candidacy emerge as central organising principles in the diagnostic pathway for suspected cancer in primary care. Legitimacy—defined here as the perceived ‘right’ of a patient’s symptoms to be taken seriously—both precedes and interlocks with candidacy, the process by which patients become recognised as candidates for further investigations. Our findings align with previous research using the candidacy framework, according to which clinicians attributed symptoms to age or health habits,^24^ and our findings add the pre-existing conditions perspective. In what follows, we first unpack how legitimacy underpins candidacy and then explore the social and moral dimensions through which pre-existing conditions, triaging steps and ‘metrics’ inform clinicians’ judgements about who is legitimate for cancer investigation.

Legitimacy functions as the gateway criterion; patients must first establish their symptoms as ‘real’ or ‘valid’ before they decide to enter the healthcare pathway. In our data, patients undergo an internal self-evaluation—’Is this legitimate enough to bother the GP?’—and only when symptoms pass this personal legitimacy test do they seek an appointment. This finding aligns well with current literature around ‘wasting doctors’ time’^15^ and explains the internal process patients go through.

Once in primary care, the triage process (by reception, by nurse or via online form) serves as a second filter: unless a patient’s presentation is deemed legitimate, they cannot progress to a face-to-face consultation. Our findings align with current research that explains the triage process in primary and pre-primary care and the challenges and dangers of over or under triaging.^25^ At the consultation itself, legitimacy continues to mediate candidacy. GPs and nurses rely on patients’ narrative clarity and consistency—precise chronology, frequency and severity—to validate their claim to further investigations. When patients articulate red-flag symptoms convincingly, they reinforce their own legitimacy and thus set their candidacy for imaging or specialist referral. Thus, legitimacy is not a one-off gatekeeper but an ongoing, dynamic judgement that enables candidacy at every stage of care. Our findings confirm previous research that highlights the importance of interpretation and management of symptoms in primary care,^26^ the problems of undisclosed symptoms during consultations,^27^ or how symptoms are labelled medically unexplained very early in the consultation because of how patients have described them or the nature of the symptom.^28^ However, unlike other research, our data reveal why the effectiveness of describing a symptom is crucial for clinicians’ decision-making.

Legitimacy is not merely a clinical abstraction; it is deeply inflected by social and moral judgements. We identify three inter-related domains in which these moral economies play out. Clinicians routinely invoke comorbidities as both a clinical and moral lens. While some view the presence of COPD or diabetes as a reason to lower the threshold for investigations, others declare that these ‘muddy the waters,’ attributing all symptoms to pre-existing conditions and thereby delaying cancer investigations.^12^ Importantly, mental health conditions—anxiety and depression—occupy a uniquely stigmatised space. Patients with these diagnoses are sometimes labelled ‘hypochondriacs,’ a moralising term that diminishes their legitimacy. This stigma compounds documented delays in cancer diagnosis for those with mental illness,^11 29^ because clinicians may discount or reattribute their somatic concerns to psychological origins, rather than considering them as red-flag symptoms.

Triage is both procedural and moral. Receptionists and nurses act as ‘street-level bureaucrats’ who must assess which patients are ‘worthy’, or legitimate, of consultation slots. Non-verbal cues—tone of voice, urgency—often substitute for clinical data in phone‐based triage, privileging those who can perform legitimate pain or fear. The importance of non-verbal cues has for long been highlighted for improving patient-centred care.^30^ Similarly, online forms favour patients with digital literacy and concise writing skills and/or well-informed statements. These social determinants of communication thus shape legitimacy and, by extension, candidacy.

Beyond narrative, clinicians draw on ‘objective’ metrics—blood tests, family history, smoking status, weight—as arbiters of legitimacy. These metrics carry moral weight: a positive family history or alarming lab result validates a patient for investigation, whereas an ‘average’ metric profile may be used to deny further work-up. Here again, legitimacy is co-constructed. Patients who can recall detailed family histories or who present with clear laboratory abnormalities are morally rewarded with legitimacy, while those who cannot are left with the moral residue of suspicion or dismissal.

From these dynamics emerge provisional characteristics of the legitimate patient—not as a positivistic or deterministic blueprint, but as a heuristic to expose and counteract the stereotypes and biases that hinder equitable care and leave patients at risk outside the care they need:

- Biological validity. Does the patient present with measurable risk factors—abnormal test results, strong family history, relevant comorbidities or symptom patterns associated with cancer—that underpins a need for further investigations?
- Effective communication. Does the patient articulate symptoms clearly, with the precision and chronology clinicians need to align the patient’s narrative with their clinical decision-making requirements?
- Perceived credibility. Are the patient’s concerns measured and seen by others as rational rather than excessive? Do the clinical team need to check they are not stereotyping the patient as ‘hypochondriac’?
- Social and moral positioning. Does the patient occupy a moral stance—through age, occupation or social comportment—that renders them legitimate of medical attention rather than burdensome?

These dimensions do not define what makes a patient legitimately in need of care, but rather reflect how legitimacy is often constructed, recognised or denied within clinical interactions. Mapping these dynamics is not a guide for profiling patients, but a tool for clinicians to recognise when implicit biases or systemic norms are disadvantaging certain individuals. By mapping patients against these dimensions, clinicians can identify when legitimate need is being overlooked—whether due to stigma around mental health, communication barriers or gaps in biometric data—and intervene with targeted equity strategies (eg, triage standardisation, anti-stigma training, inclusive risk tools). Characteristics like these could be used to tackle stereotypes and biases and build personas that could allow equal and inclusive care.^31^

Taken together, our findings illustrate how legitimacy enables candidacy through interwoven clinical, social and moral logics. Legitimacy precedes candidacy in that it is the necessary condition for entering the diagnostic pathway, but it is also reciprocally reinforced as candidacy progresses: the more a patient ‘performs’ legitimacy through effective narrative, triage compliance or objective metrics, the stronger their candidacy becomes.

The question, thus, of whether pre-existing conditions affect cancer diagnosis in primary care does not have a straightforward answer. Instead, it raises broader concerns about the processes and criteria through which legitimacy is constructed, evaluated and acted on. While this study provides an in-depth investigation of how pre-existing conditions shape cancer diagnosis in primary care, some limitations should be acknowledged. The research did not analyse patients’ and clinicians’ data based on demographic variables such as age, gender or socioeconomic deprivation. As the primary focus was on how specific pre-existing conditions influence cancer diagnosis, interviews concentrated on these conditions as distinct nosological entities with unique risk profiles. Another limitation is the lack of comparison with patients without pre-existing conditions, which would have provided a broader understanding of decision-making processes in primary care. Finally, a further limitation of this article is the lack of reporting on the outcomes of the consultations. This gap arose for several reasons: (a) in many cases, the diagnostic process was still ongoing at the time of the interviews; (b) clinicians tended to reflect on past consultations with an emphasis on the decision-making process rather than the outcomes and (c) although some patients were referred for further investigations, they often focused in the interviews on the time it took to receive a referral rather than the referral itself.

Having interviewed patients and clinicians across England, our research highlights critical issues within primary care and the complex journey that patients with pre-existing conditions undertake when seeking cancer. Understanding the role of legitimacy in these processes offers valuable insights into how healthcare access and clinical decision-making are shaped, ultimately informing strategies to improve patient outcomes. Our findings indicate that legitimacy does not only refer to patients with pre-existing conditions and potential cancer diagnosis in primary care but also contributes to the understanding of help-seeking behaviours for other conditions, contrasting, for instance, those whose diagnosis can be based on more subjective information (mental health, functional symptoms, etc).

One of the most crucial findings of our research is the importance of how effectively symptoms are described during consultations. We recommend that further research should explore clinicians’ expectations of patients, as well as patients’ health literacy and how it is communicated and interpreted during the consultation. Further research should also explore how legitimacy is influenced by patient demographics such as gender, ethnicity, age and socioeconomic status. Understanding whether certain groups disproportionately fail to meet tacit legitimacy thresholds could help explain disparities in early cancer diagnosis. Also, including a control group of patients without pre-existing conditions would help to isolate the effects of comorbidities on legitimacy judgments and candidacy outcomes. Future research could expand the exploration of how clinician age, experience and speciality training influence thresholds for investigation. Finally, research is needed on how digital triage tools encode or mitigate moral judgements embedded in legitimacy assessments. Do algorithms reduce bias, or reproduce it in new ways?

Recommendations occurring from this research entail the development and implementation of nationally consistent triage criteria—including for phone, online and in-person formats—that focus on clinical risk rather than subjective impressions. This could reduce bias introduced by non-clinical gatekeepers and communication disparities. Another recommendation is interventions on mental health stigma, disability bias and the social determinants of help-seeking behaviour for all primary care staff, including receptionists. Finally, patient personas could be used to leverage the ‘provisional profile of the legitimate patient’ as a teaching tool in GP education, helping clinicians identify when cognitive biases or moral judgments may be influencing their diagnostic decisions.

## Supplementary material

10.1136/bmjopen-2025-103288 online supplemental file 1

10.1136/bmjopen-2025-103288 online supplemental file 2

## Data Availability

No data are available.
