# Supplementary material for: How do primary care consultation dynamics affect the timeliness of cancer diagnosis in people with one or more long-term conditions? A qualitative study
Source: BMJ Open. 2025 Sep 28;15(9):e103288. doi: 10.1136/bmjopen-2025-103288 (PMC12481337; doi:10.1136/bmjopen-2025-103288)
Supplement: online supplemental file 1 [file bmjopen-15-9-s001.docx]

***Help us to spot cancer early: An interview study to explore your experiences***

**TOPIC GUIDE – GPs & NURSES**

*(Confirm consent)*

**Define context: in WP2 we investigate five conditions (diabetes, obesity, COPD, anxiety/depression, multimorbidity)**

1. How do you think comorbidities affect possible cancer diagnosis in primary care?

***PROMPT:*** *Obstacle/facilitator: which category acts like obstacle/facilitator and why*

1. How would you say that patients with comorbidities tend to respond to a change in their symptoms or a new symptom? Give examples, e.g., diabetes

***PROMPT:*** *self-manage symptoms, over-reaction/hypochondria, denial, extreme anxiety. Are there differences between conditions [e.g., diabetes, COPD etc.]?*

1. Symptom attribution: when patients visit you with new or persistent symptoms, what explanations do they give about their symptoms? Are there differences between conditions?

***PROMPT:*** diabetes and medication (patients attribute abdominal symptoms, eg change in bowel habits, to medication)

1. Can you describe any factors that affect the way patients communicate their symptoms or things they are worried about?

***PROMPT:*** *Socioeconomic/education differences, ability to spot and describe symptoms*

1. Does comorbidity (or any other factor, e.g., age) affect your decision to refer a patient for further tests/investigations? [Nurses may refer a patient to a GP]. Prior odds, alternative explanations, surveillance

***PROMPT:*** *When and why might you adopt a watch and wait approach? When and why might you arrange regular appointments?*

1. If you know the patient, does it affect the process of decision making? In what ways?

***PROMPT:*** *Do you think continuity of care is important?*

1. How do you know when a symptom is of the pre-existing condition and not of cancer?
2. Do you treat each condition separately or multiple conditions together?
3. When do you get alerted to a symptom? Signal to noise

***PROMPT:*** *Examples, symptoms that may be confused with drug effects (e.g. diabetes and medication)*

1. How do you deal with the situation where a patient says one of their symptoms is bothering them the most, but you are more concerned by another? Competing demands

***PROMPT:*** *consultation time, shared decision-making*

1. In what ways, if any, do guidelines/protocols help you in decision-making for possible cancer diagnosis?
2. Do you have any other comments about treating/diagnosing/referring patients with comorbidities?
