# Supplementary material for: How do primary care consultation dynamics affect the timeliness of cancer diagnosis in people with one or more long-term conditions? A qualitative study
Source: BMJ Open. 2025 Sep 28;15(9):e103288. doi: 10.1136/bmjopen-2025-103288 (PMC12481337; doi:10.1136/bmjopen-2025-103288)
Supplement: online supplemental file 2 [file bmjopen-15-9-s002.docx]

**Help us to spot cancer early: An interview study to explore your experiences**

**TOPIC GUIDE – PATIENTS**

*(Confirm consent; ask patient about friends/family/other sources of support)*

**STAGE 1 Pre-existing condition/comorbidity**

1. Please, will you tell me a little bit about the pre-existing condition(s) you have – in terms of symptoms, frequency of visiting the GP/nurse, how it affects your life?
2. Please, will you describe a typical appointment with a GP/nurse?

**PROMPT**: reasons for visiting the GP, is it important to see the same GP, confidence in the GP/nurse, access issues/special requirements

**STAGE 2 Process of deciding to visit the GP**

1. Please, will you tell me, what were the changes in your body that made you decide to (try to) book an appointment with the GP/nurse?
   1. Symptom attribution: what did you first think of the symptom? What did you think it was? (eg., side-effects of meds i.e., metformin etc.)

**PROMPT**: look for first reactions to the symptoms and any self-management practices (e.g., Internet research), how and why did they decide to visit the GP - intensity, duration of symptoms, did the symptoms make them feel uncomfortable?

Ask patient to think about a particular appointment (in the past 6 months), about a particular symptom/s.

How did your existing condition(s) affect your decision to seek help? How did you existing condition(s) affect how you interpreted the symptoms?

1. Did your family/friends influence you to visit the GP/nurse – how did they do this and to what extent?

**PROMPT**: did anyone notice the symptoms? Did s/he discuss it with anyone? Have family/friends taken the initiative to talk to her/him?

1. Please, will you tell me, why did you decide to (try to) book a quick appointment? Or why did you delay booking an appointment?

**PROMPT**: explore worries, hypochondria, over-reassurance from previous tests, denial, COVID, fear, family/caring/financial factors, rationing use of NHS/practice

**STAGE 3 The appointment and relationship with the GP/nurse**

1. At the appointment, how did you describe your symptoms?

**PROMPT**: explore language used. How did you existing condition(s) affect how you describe your symptom/s?

1. Please, will you tell me, how did you choose which symptoms to talk about with your GP?

**PROMPT:** How did you prioritise the symptoms to talk about?

1. How did the GP/nurse explain your symptoms?

**PROMPT**: any worries or thoughts of cancer?

1. Please, will you describe what happened as a result of your appointment?

**PROMPT**: referral for tests? Advised to make another appointment? How long did it take? Decision to watch and wait?

1. Looking back at the appointment you just described, please will you tell me how the decisions were made?

**PROMPT**: shared decision making about referrals for tests/investigations? Did the GP take patient preference into account, did they discuss reasons for referral?

1. How do you get on with your/the GP/nurse?

**PROMPT**: time given from the GP to describe her/his worries etc., pre-existing condition and effect on the process, importance (or not) of seeing same GP/nurse

**STAGE 4 After the appointment**

1. Please, will you tell me, what has happened since the appointment?
2. Please, will you describe how you feel about what happened during and after the appointment

**PROMPT**: were your needs covered? Did you feel rushed, alarmed, reassured? Still undergoing tests/investigations?

1. What do you think might have happened if you haven’t made the appointment?

**PROMPT**: was it the right decision to have the appointment with the GP/nurse? Why is this?

1. What advice would you give to a friend/family member if they had the same experience (symptoms) as you?

**Help-seeking (general)**

*Thank you for talking to me about the specific appointment you had with your GP / practice nurse. I’d now like to ask you about your decision to seek help about your symptoms in a more general way.*

Signal to noise (symptom recognition)

1. As someone with a known health condition(s), you sometimes might get a new symptom, how do you know whether this is part of the same health problem or not
2. Tell me more about how you know which symptoms belong to your health condition(s).

Perceived prior odds

1. Given that you have one or more pre-existing conditions how does that affect your decision to seek help for a new symptom?
2. Tell me more about how your knowledge of your existing health condition(s) makes you more, or less, likely to consider the new symptom as part of the same health problem(s).

Competing demands

1. In what ways do you make sense of your ‘new’ symptom when you are experiencing multiple conditions/symptoms all of which take your time and attention?
2. How do you deal with the situation where you as the patient says one of your symptoms is bothering you the most, but the doctor or nurse seem more concerned by another?

Alternative explanations (attribution theory)

1. In what ways do you seek to find an explanation for your symptom?
2. Tell me more about how your symptom can be explained by your on-going health condition(s) versus whether it is a symptom of something new?

Surveillance

1. How often do you visit the GP to check the condition?
   1. How often would you like to visit the GP? Is this enough?
2. How often does the GP want to do a blood test/scan etc.?
