# Supplementary material for: How do primary care consultation dynamics affect the timeliness of cancer diagnosis in people with one or more long-term conditions? A qualitative study
Source: BMJ Open. 2025 Sep 28;15(9):e103288. doi: 10.1136/bmjopen-2025-103288 (PMC12481337; doi:10.1136/bmjopen-2025-103288)
Supplement: Supplementary file 3 [file bmjopen-2025-103288.reviewer_comments.pdf]

# PEER REVIEW HISTORY

BMJ Open publishes all reviews undertaken for accepted manuscripts. Reviewers are asked to complete a checklist review form and are provided with free text boxes to elaborate on their assessment. These free text comments are reproduced below.

## ARTICLE DETAILS

### Title (Provisional)

How do primary care consultation dynamics affect the timeliness of cancer diagnosis in people with one or more long-term conditions? A qualitative study

### Authors

Valasaki, Maria; Carter, Mary; Winder, Rachel; Shephard, Elizabeth; Valderas, Jose M.; Merriel, Samuel William David; Farmer, Leon; Summers, Beccy; Dean, Sarah Gerard; Morgan-Trimmer, Sarah

---

## VERSION 1 - REVIEW

---

| | |
|---|---|
| **Reviewer** | **1** |
| **Name** | **van der Wardt, Veronika** |
| **Affiliation** | **University of Marburg, Department of Primary Care** |
| **Date** | **22-May-2025** |
| **COI** | **None** |

---

Many thanks for this well written and important paper. Introduction, while a bit lengthy, and method section is good. I would recommend to add an assessment of trustworthiness.

The Results section needs tightening up. There are repetitions (e.g. in the section about hypochondriacs or that legitimacy is important for decision making) and points could be made with much more focus. In addition, you mention that many participants had more than one condition but the table at the top of the Results section only shows one. Please clarify. It also would be interesting to see if gender of GP and/or patient played a role in the perception of legitimacy. If you think the data would allow useful findings, please add those, if not, add it as a limitation in the discussion. The conclusion at the end of the Discussion sections should lead to more interesting applications of the results for patient care and research.

---

| | |
|---|---|
| **Reviewer** | **2** |
| **Name** | **Nemlander, Elinor** |

**Affiliation** **Karolinska Institutet, Division of Family Medicine and Primary Care, Department of Neurobiology, Care Sciences and Society**

**Date** **31-May-2025**

**COI** **None**

---

General comments

This manuscript addresses a highly relevant and timely topic: how the dynamics of primary care consultations may shape the timeliness of cancer diagnosis in people living with one or more long-term conditions. The use of interviews with both patients and primary care professionals adds valuable depth and allows for a nuanced exploration of perceptions and relational processes within consultations.

The study offers rich empirical material, and the central theme of legitimacy is potentially a very useful lens for understanding subtle barriers to diagnostic escalation. I appreciate the authors' efforts to explore the social and moral dimensions of help-seeking and triage, which are often difficult to capture.

That said, I believe the manuscript would benefit from revision and clarification in several key areas. In particular, greater methodological transparency and a more precise conceptual framing of legitimacy would strengthen the overall contribution of the paper.

Major comments

1. Scope and contribution

The article suggests that long-term conditions influence how patients are assessed and managed in relation to possible cancer symptoms. However, the lack of a comparator group (e.g. patients without long-term conditions) makes it difficult to determine which findings are specific to long-term conditions. I encourage the authors to more clearly position the study within the existing literature on diagnostic delay and complexity in consultations, and to state explicitly what new contribution this study offers.

2. Study design and methods

It would be helpful to clarify the interview process in more detail:

– Were all interviews individual, or were any conducted in groups?

– How many researchers conducted the interviews, and what were their professional backgrounds or prior assumptions?

– Was a topic guide used, and if so, could it be summarised or included as supplementary material?

These clarifications are particularly important given the study's reliance on reflexive thematic analysis, where interviewer positioning and analytical reflexivity are central.

3. Analysis process

The description of the thematic analysis could be expanded. For example:

– How were the codes developed and refined?

– How many team members were involved in coding, and how were disagreements handled?

4. Presentation of findings

While the findings are interesting and often compelling, they could be presented more concisely. There is some repetition across the subthemes, and the manuscript is quite long in its current form. A summary table outlining themes and subthemes could help guide the reader and improve readability.

In addition, the key assertion, that perceptions of legitimacy are central to whether patients are escalated within the system, would benefit from further empirical grounding. The link between these perceptions and actual clinical outcomes is often implied, but not always clearly demonstrated in the data.

5. Framing of 'legitimacy'

The term legitimacy is used in multiple ways, self-perception, professional assessment, as a social or moral construct, which makes its role somewhat difficult to pin down. A clearer, more consistently applied definition would help the reader follow the argument.

The detailed outline of characteristics forming a "profile of the legitimate patient" reflects many of the clinical cues that understandably inform risk assessments in primary care. That said, the framing as a 'profile' may still unintentionally suggest a fixed or ideal patient type. To avoid this, it might help to further emphasise the interpretive and critical intent of this categorisation, and perhaps reflect on patients who may fall outside of this construct yet remain at risk. This would reinforce the value of the framework without risking essentialising patient presentations.

Minor points

Please clarify the total number of interviews, as there appears to be a discrepancy between the text and figure.

Consider tightening the manuscript by streamlining sections that are conceptually or thematically overlapping.

The reporting of ethics and funding is clear and complete.

Recommendation: Major Revision

This is a thoughtful and well-intentioned study that raises important questions about how patient narratives are evaluated in complex primary care contexts. With revisions to clarify methods, sharpen conceptual focus, and streamline the presentation, the manuscript has the potential to make a strong contribution. I hope the authors will find these comments constructive and useful in further developing their work.

## VERSION 1 - AUTHOR RESPONSE

| | Comments | Revision |
|---|---|---|
| Reviewer 1 | Introduction, while a bit lengthy, and method section is good. | Thank you. However, taking into consideration reviewer's 2 comments, we have shortened both sections. |
| | I would recommend to add an assessment of trustworthiness. | Added in paragraph 3 of the Analysis. |
| | The Results section needs tightening up. There are repetitions (e.g. in the section about hypochondriacs or that legitimacy is important for decision making) and points could be made with much more focus. | Thank you for the comments. Results are tightened up and repetitions are removed. We have added a table with illustrative quotations and have a more cohesive text. |
| | In addition, you mention that many participants had more than one condition but the table at the top of the Results section only shows one. | This comment has been removed for clarity. While our participants were initially recruited through general practices based on records indicating one of the target conditions (e.g., diabetes), during the interviews they often reported additional conditions, such as fibromyalgia. Although we initially considered it interesting to report this, upon reviewing the text in light of your comment, we recognised that it introduced unnecessary complexity. Therefore, we decided to remove it to maintain clarity. |
| | It also would be interesting to see if gender of GP and/or patient played a role in the perception of legitimacy. If you think the data would allow useful findings, please | Unfortunately, we did not analyse data based on gender and age. Although we do have some |

| | | |
|---|---|---|
| | add those, if not, add it as a limitation in the discussion. | implications, we do not have data supporting this. However, we did add this limitation in the discussion. |
| | The conclusion at the end of the Discussion sections should lead to more interesting applications of the results for patient care and research | We have amended the end of the discussion with a more detailed consideration for implementing the research findings and further research |
| **Reviewer 2** | The article suggests that long-term conditions influence how patients are assessed and managed in relation to possible cancer symptoms. However, the lack of a comparator group (e.g. patients without long-term conditions) makes it difficult to determine which findings are specific to long-term conditions. I encourage the authors to more clearly position the study within the existing literature on diagnostic delay and complexity in consultations, and to state explicitly what new contribution this study offers. | Unfortunately, we did not have a comparator group. Introduction and discussion are revised and more relevant literature, as suggested (diagnostic delay and complexity in consultations), is added. Contribution of this study is hopefully clearer this time. |
| | – Were all interviews individual, or were any conducted in groups?<br>– How many researchers conducted the interviews, and what were their professional backgrounds or prior assumptions?<br>– Was a topic guide used, and if so, could it be summarised or included as supplementary material? | -All interviews were individual, and now is clarified in the text<br>-2 researchers conducted the interviews with non-clinical background and experience in qualitative research. This information is now added in the text.<br>-Two topic guides were used, and are attached as supplemental material |
| | – How were the codes developed and refined?<br>– How many team members were involved in coding, and how were disagreements handled? | -Description added in 3rd paragraph of the analysis<br>-Fortunately, there were no disagreements, but there was reflective discussions within the team and during meetings with PPIE members. Relevant |

| | | |
|---|---|---|
| | | information is included in the Analysis section. |
| | While the findings are interesting and often compelling, they could be presented more concisely. There is some repetition across the subthemes, and the manuscript is quite long in its current form. A summary table outlining themes and subthemes could help guide the reader and improve readability. | Changes made: we avoided repetitions and removed the existing ones, we added a table with illustrative quotations, and have shortened the text. |
| | In addition, the key assertion, that perceptions of legitimacy are central to whether patients are escalated within the system, would benefit from further empirical grounding. The link between these perceptions and actual clinical outcomes is often implied, but not always clearly demonstrated in the data. | Indeed, this is accurate; however, the interviews were designed to focus on the decision-making process rather than the outcomes of consultations. We have acknowledged this in the limitations section and provided explanations for why outcome data were not consistently captured. |
| | The term legitimacy is used in multiple ways, self-perception, professional assessment, as a social or moral construct, which makes its role somewhat difficult to pin down. A clearer, more consistently applied definition would help the reader follow the argument. | Although legitimacy comes up as a complex concept, we tried to provide a consistent and clearer, this time, term of legitimacy as emerged from our data. |
| | The detailed outline of characteristics forming a “profile of the legitimate patient” reflects many of the clinical cues that understandably inform risk assessments in primary care. That said, the framing as a ‘profile’ may still unintentionally suggest a fixed or ideal patient type. To avoid this, it might help to further emphasise the interpretive and critical intent of this categorisation, and perhaps reflect on patients who may fall outside of this construct yet remain at risk. This would reinforce the value of the framework without risking essentialising patient presentations. | That is a very good point, and we have now tried to clarify this in the suggested way. |

| | | |
|---|---|---|
| | Please clarify the total number of interviews, as there appears to be a discrepancy between the text and figure. | Clarified |
